# Apical myocardial infarction with bizarre coronary images mimicking left ventricular apical ballooning syndrome: a case report

**DOI:** 10.1186/1752-1947-8-124

**Published:** 2014-04-09

**Authors:** Tetsuya Nomura, Natsuya Keira, Shunta Taminishi, Hiroshi Kubota, Yusuke Higuchi, Sho Ikegame, Kensuke Terada, Taku Kato, Yota Urakabe, Tetsuya Tatsumi

**Affiliations:** 1 Department of Cardiovascular Medicine, Nantan General Hospital, 25, Yagi-Ueno, Yagi-cho, Nantan City, Japan

**Keywords:** Apical myocardial infarction, Coronary vasospasm, Left ventricular apical ballooning syndrome, Spontaneous coronary artery dissection

## Abstract

**Introduction:**

Although several etiopathogenetic mechanisms have been proposed, the causes of left ventricular apical ballooning syndrome are still controversial.

**Case presentation:**

A 51-year-old Japanese woman consulted the emergency room complaining of the sudden onset of anterior chest pain while shopping. We initially suspected her disease as left ventricular apical ballooning syndrome based on her clinical background and laboratory examinations. However, the initial coronary angiogram demonstrated diffuse lesions in her distal left anterior descending coronary artery, and she was finally diagnosed with apical myocardial infarction. The blood flow in her distal left anterior descending coronary artery had markedly improved in the chronic phase. If the reduced blood flow in her distal left anterior descending coronary artery was induced by coronary vasospasm and the vasospasm was relieved before the coronary angiogram was performed, this case must be diagnosed as left ventricular apical ballooning syndrome.

**Conclusion:**

We think this case may promote discussion regarding the pathophysiology of left ventricular apical ballooning syndrome.

## Introduction

Left ventricular apical ballooning syndrome (LVBS), also known as takotsubo cardiomyopathy, is an emerging clinical entity presenting with acute chest pain or dyspnea, associated with new ST-T segment elevation mimicking ST-elevated myocardial infarction (STEMI). Among patients presenting with clinically suspected acute coronary syndrome, its prevalence is reported to range from 1.2 to 2.0% and it occurs almost exclusively in postmenopausal women, often being associated with emotional or physical stress [1,2]. Although several etiopathogenetic mechanisms have been proposed, the causes of LVBS are still controversial.

We encountered a case of initially suspected LVBS based on the clinical background. However, coronary angiography (CAG) demonstrated bizarre images of the left anterior descending (LAD) coronary artery. Although the final diagnosis was apical myocardial infarction induced by prolonged reduced blood flow in the distal portion of the LAD coronary artery which extended around the apex, this case may promote discussion regarding the pathophysiology of LVBS considering the specific clinical course.

## Case presentation

**Figure 1:**
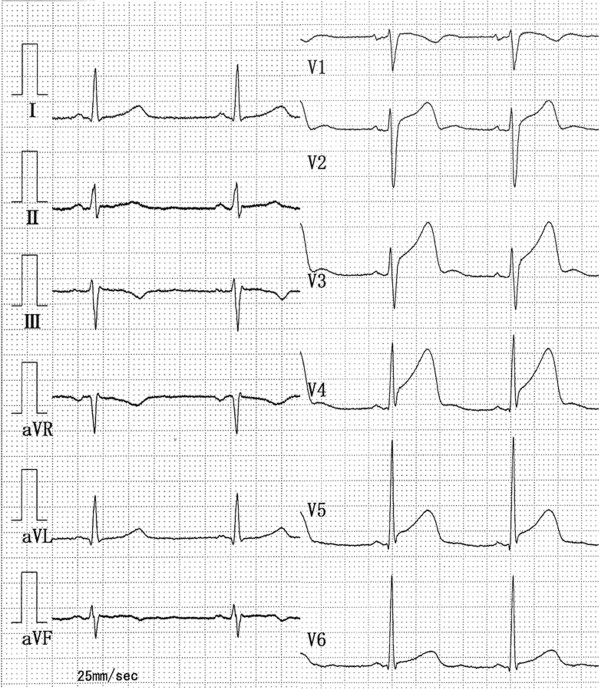
A 12-lead electrocardiogram in the emergency room demonstrated an increased ST-segment in V2 to V5 anterior chest leads.

A 51-year-old Japanese woman (body mass index = 21.0) with no medical history consulted the emergency room complaining of the sudden onset of anterior chest pain while shopping. She had no coronary risk factors. Her blood pressure was 192/100mmHg, and pulse was 64/minute and regular. A 12-lead electrocardiogram demonstrated an increased ST-segment in V2 to V5 anterior chest leads (Figure 1), and echocardiography showed akinesis localized in her left ventricular apex. A laboratory study showed an increased level of white blood cells (10,480/μL) and a positive troponin T. Although there was no apparent triggering of the episode such as emotional or physical stress, we suspected LVBS based on the findings of persistent anterior chest pain in a postmenopausal woman with no coronary risk factors and left ventricular wall motion asynergy localized in the apex.

**Figure 2:**
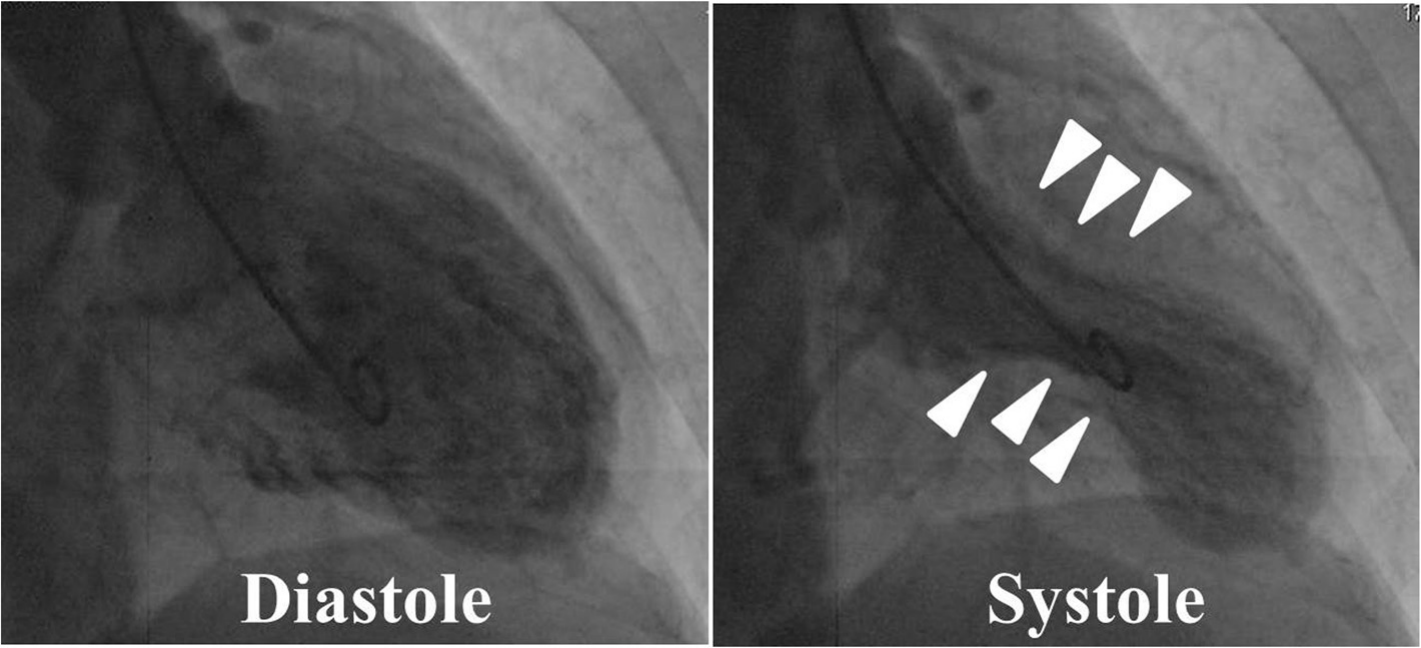
A left ventriculogram demonstrated akinesis in the apex and hyperkinesis in the basal area (arrow heads) of the left ventricle that closely resembled typical findings of left ventricular apical ballooning syndrome.

**Figure 3:**
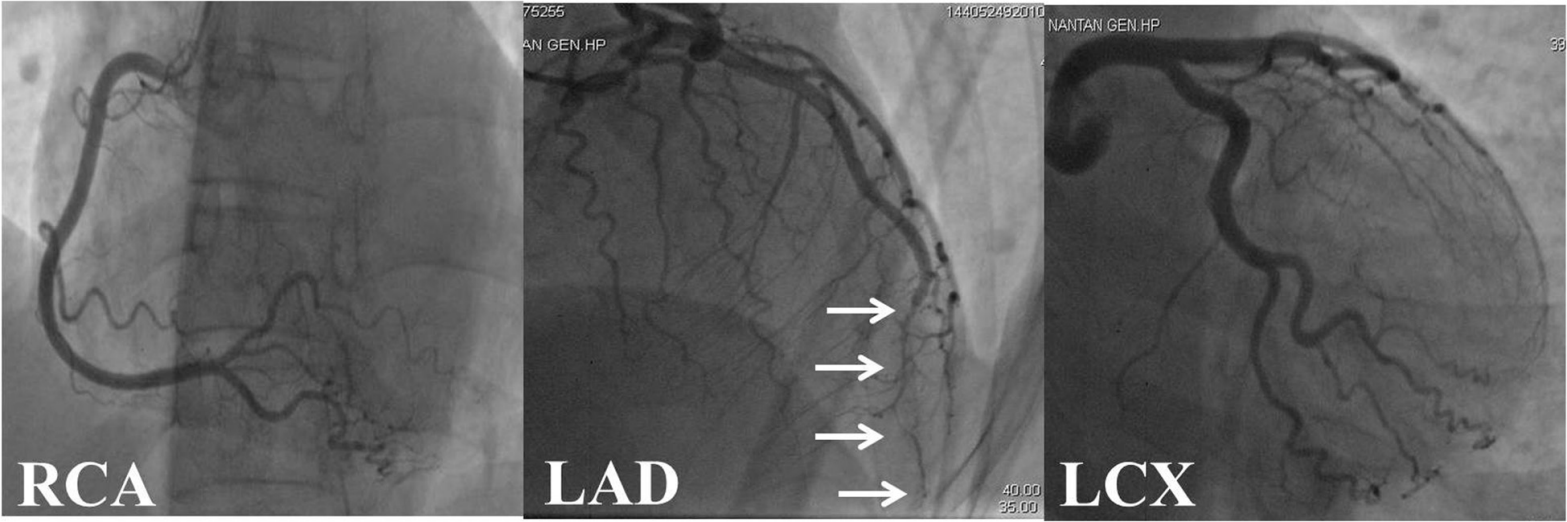
**Right coronary artery and left circumflex showed no abnormal findings.** The distal left anterior descending artery looked like a withered branch (arrows). Abbreviations: LAD, left anterior descending; LCX, left circumflex; RCA, right coronary artery.

Therefore, we performed emergency cardiac catheterization. Left ventriculography demonstrated akinesis in the apex and hyperkinesis in the basal area of her left ventricle, which closely resembled the typical findings of LVBS (Figure 2). Next, we performed CAG. Her right coronary artery and left circumflex artery showed no abnormal findings (Figure 3). However, the distal portion of her LAD artery looked like a withered branch (arrows in Figure 3). Because we considered the condition to have been induced by coronary vasospasm, we injected a total of 3mg of isosorbide dinitrate (ISDN) directly into her left coronary artery, but no change in her LAD artery appearance was observed. We did not try to perform any other imaging modalities, such as an intravascular ultrasonography (IVUS) or an optical coherence tomography (OCT) for fear that those devices might be invasive to coronary lesions. At that time, because her chest symptom had mostly resolved and her vital signs had stabilized, we completed cardiac catheterization.

We started continuous intravenous infusion of nicorandil at a rate of 2mg/hour and oral administration of aspirin and calcium blockers. Creatine phosphokinase was maximal at 1073mg/dL the next day. A Tc-pyrophosphate cardiac scintigram revealed an increased uptake of the radioisotope localized at the left ventricular apex. Based on her clinical course, she was diagnosed with apical myocardial infarction and discharged on day 8 of hospitalization after finishing cardiac rehabilitation.

**Figure 4:**
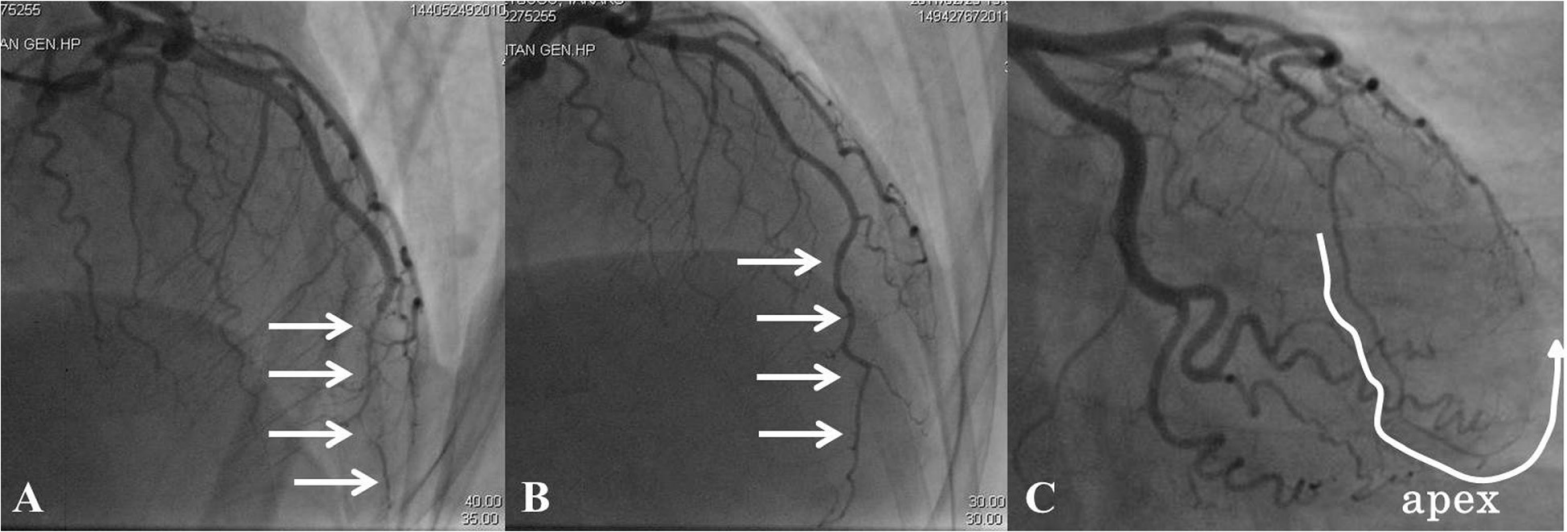
**Follow-up coronary angiography was performed after 5 months.** The left anterior descending artery extended around the apex **(C**; highlighted with white line**)**, and blood flow in its distal portion (arrows) had markedly improved **(B)** compared with that (arrows) in acute phase **(A)**.

Follow-up echocardiography was undertaken twice per month post-discharge; her left ventricular apex remained hypokinetic throughout. This finding is discrepant with LVBS whereby the wall motion defect is reversible. After 5 months from the acute phase, she received follow-up CAG (Figure 4B,C). It was found that her LAD coronary artery had extended around the apex (Figure 4C), and blood flow in the distal portion of her LAD artery had normalized compared with that in the acute phase (Figure 4A). We suggest that improvement of her LAD artery appearance might be derived from remission of the coronary vasospasm or the natural healing of spontaneous coronary artery dissection (SCAD), and the prolonged reduced blood flow in the LAD artery in this case resulted in apical myocardial infarction mimicking LVBS.

## Discussion

LVBS is not a rare clinical entity and presents with acute chest symptoms with new ST-T segment elevation mimicking STEMI. Regional transient myocardial dysfunction is observed, typically localizing at the left ventricular apex and usually extends beyond a single vessel territory. Minimum serum cardiac enzyme release and the absence of organic lesions or spasm of coronary arteries are seen [3]. Although several etiopathogenetic mechanisms have been proposed, such as multivessel epicardial vasospasm [4,5], catecholamine-induced myocardial stunning [6,7], spontaneous coronary thrombus lysis [8], and acute microvascular vasospasm [9-11], the causes of LVBS are still being debated.

LAD artery obstruction, resolved within a few hours of the onset of symptoms (due to remission of spasm or spontaneous thrombolysis) before an angiographic study is conducted, is one of the hypotheses for the pathophysiology of LVBS. Spontaneous early reperfusion can explain phenomena such as minimal enzymatic release and left ventricular stunning rather than necrosis [8]. One argument against this hypothesis includes the observation that the extent of left ventricular dysfunction in LVBS cannot be explained by the blood-supplying area of a single coronary artery [12]. For this argument, it is proposed that a transient ischemic event in the anatomically variable LAD artery with a long course around the left ventricular apex can cause left ventricular asynergy similar to LVBS [13]. However, this is not a consistent finding in all reported LVBS cases.

We found that our patient’s distal LAD artery looked like a withered branch in the acute phase and its appearance was markedly improved in the chronic phase. For this pathophysiology, we have to make a differential diagnosis between coronary vasospasm and SCAD [14,15]. SCAD usually predominates in patients younger than this case. It is said that most people who develop SCAD have no obvious risk factors. However, several conditions may increase the risk of SCAD, including blood vessel abnormalities, inherited connective disorders, vasculitis, coronary vasospasm, systemic hypertension and others. We did not identify any predisposing factors for SCAD in this patient. The typical appearance on angiography of SCAD is that of a radiolucent line that represents the intimal medial flap, separating the flow between the true and false lumens, which seems to be different from the angiographical appearance in this case. However, an intimal flap in SCAD is not always visualized. The fact that the intracoronary injection of ISDN did not work at all to resolve the coronary flow suggests a reduced possibility of coronary vasospasm involvement. Because we did not check the lesions either with IVUS or OCT, we cannot conclude that this pathophysiology was present.

Moreover, because CAG was not evaluated until 5 months later, we have no idea about the process of recovery of LAD blood flow. If the reduced blood flow in the distal LAD artery was induced by coronary vasospasm and the vasospasm was relieved before CAG was performed, this case must be diagnosed as LVBS. Although this mechanism is only one hypothesis about the cause of LVBS, as is also presented in several previous reports, we think this case is very rare and has implications for investigating the etiology of LVBS in the future.

## Conclusions

This case report describes a case of apical myocardial infarction with bizarre initial images of LAD coronary artery mimicking LVBS. There have been proposed several etiopathogenetic mechanisms, but the causes of LVBS are still controversial. We think that this case may promote discussion on the pathophysiology of LVBS.

## Consent

Written informed consent was obtained from the patient for publication of this case report and accompanying images. A copy of the written consent is available for review by the Editor-in-Chief of this journal.

## Abbreviations

CAG: Coronary angiography; ISDN: Isosorbide dinitrate; IVUS: Intravascular ultrasonography; LAD: Left anterior descending; LVBS: Left ventricular apical ballooning syndrome; OCT: Optical coherence tomography; SCAD: Spontaneous coronary artery dissection; STEMI: ST-elevated myocardial infarction.

## Competing interests

The authors declare that they have no competing interests.

## Authors’ contributions

TN is the primary author of this paper. TN and ST took care of the patient. NK, ST, HK, YH, SI, KT, TK, YU and TT made substantial editorial revisions to the manuscript. NK made major contributions to the conception and design. All authors read and approved the final manuscript.
